# Supplementary material for: Brain anatomy of the Cambrian fossil Jianfengia multisegmentalis informs euarthropod phylogeny
Source: Nat Commun. 2025 Aug 28;16:7938. doi: 10.1038/s41467-025-62849-w (PMC12394709; doi:10.1038/s41467-025-62849-w)
Supplement: Supplementary file 1 — Supplementary Information [file 41467_2025_62849_MOESM1_ESM.pdf]

Brain anatomy of the Cambrian fossil *Jianfengia multisegmentalis* informs euarthropod phylogeny

Nicholas J. Strausfeld, David R. Andrew, Xianguang Hou and Frank Hirth

Supplementary Information

This file includes:
Supplementary Figures 1-4
Supplementary Tables 1, 2

Supplementary Figures

Contents comprise:

Supplementary Figure 1. The great appendages of Jianfengia.
Supplementary Figure 2. Jianfengia compound eye and putative cone cell prolongations.
Supplementary Figure 3. Exposure of trace neuropils and tracts in specimen YKLP11367.
Supplementary Figure 4. Inferred evolutionary relationships support Jianfengia as sister to all Mandibulata.

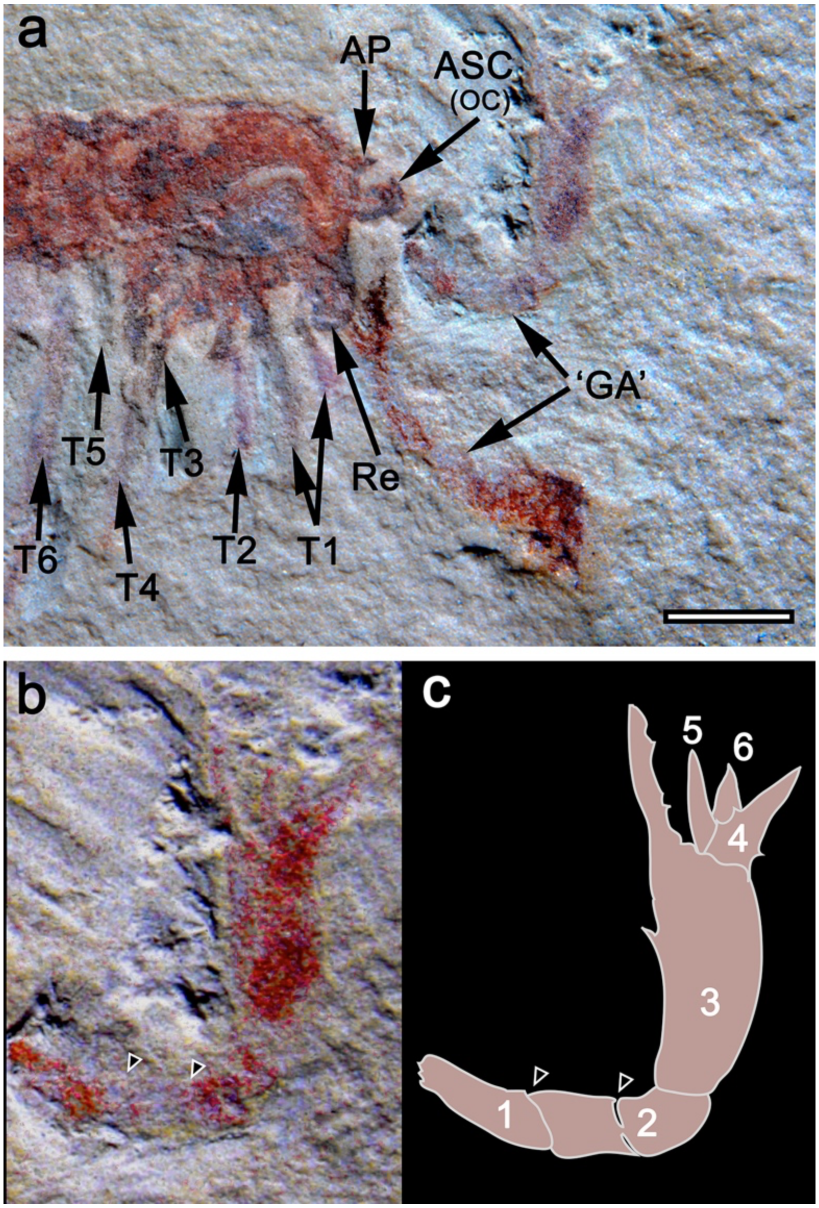

Supplementary Figure 1. The great appendages of *Jianfengia*. (**a**) Lateral view of anterior appendicular arrangements of specimen NIGPAS100123b. 'GA' indicates right and left 'great appendages.' The right compound eye (Re) is preserved; the left eye is buried in the matrix. The left anterior process (AP) is visible, its contralateral counterpart is obscured by matrix. The anterior sclerite carrying the ocelli [ASC (OC)] is visible. Both left and right biramous appendages of segment (T1) are in the same plane, whereas only the right appendages of segments T2-T4 are visible extending from beneath the carapace. (**b, c**) Isolated left GA showing podomeres (1, 2). Triangles indicate possible folds or sutures, which suggest flexibility within podomere 2. Scale bar for panels a-c=2mm.

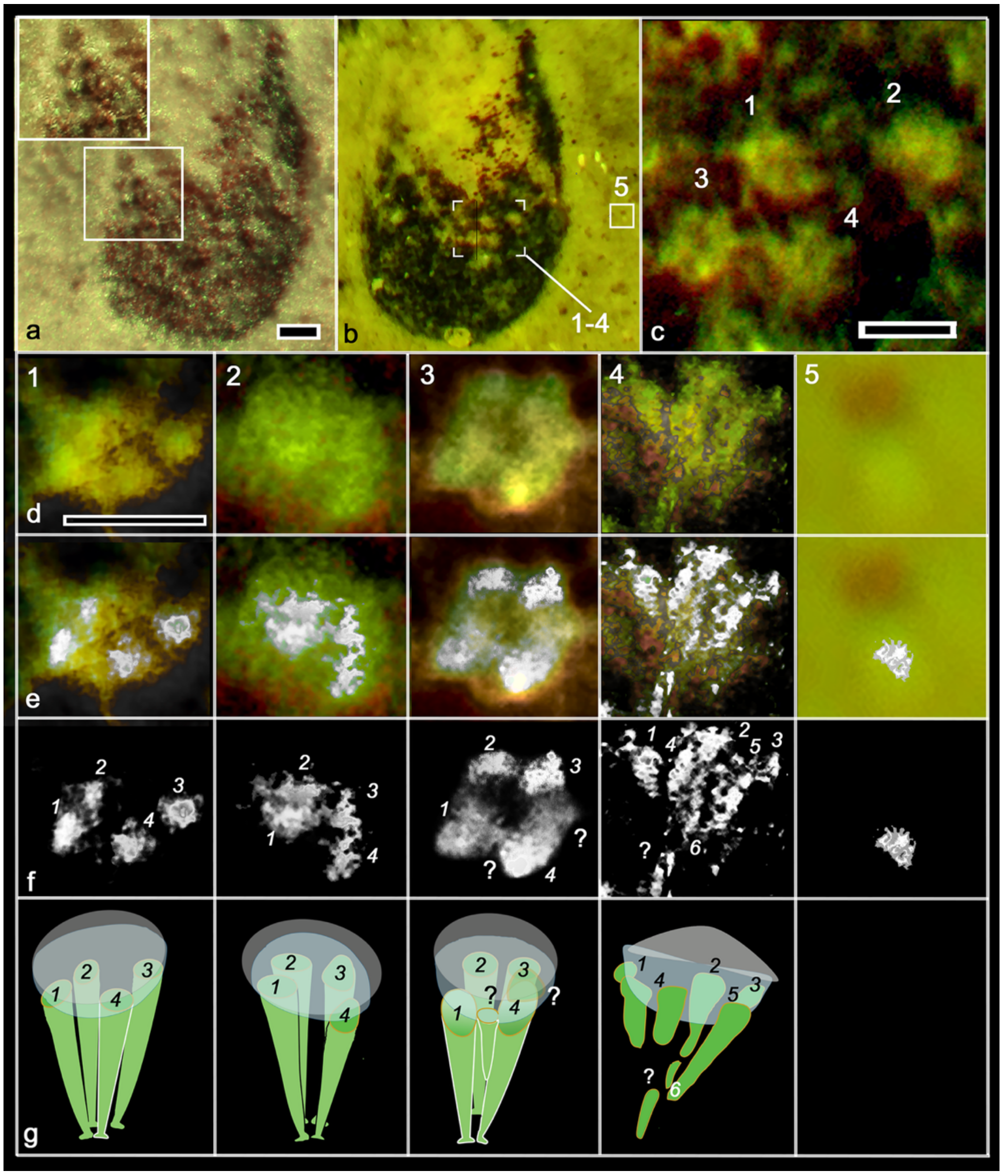

Supplementary Figure 2. *Jianfengia* compound eye and putative cone cell prolongations. (**a**) The compound eye of specimen YKLP11117 comprises approximately 120 facets some of whose convex lenses (enlarged upper left) indicates a hexagonal arrangement. (**b, c**). The bracketed area in panel b indicates four ommatidia whose lenses have been lost, thus open to illumination at the assumed level of their cones (1-4 enlarged in panel c). Control area 5 in panel b indicates one of several bright areas of the matrix sampled to exclude spurious taphonomic-generated patterns. (**d1-4**) Enlarged images within ommatidia 1-4 in panel C. (**e1-4**) Images overlain by their computed maximum intensity levels. (**f1, 2**) Maximum intensities alone demonstrate four profiles in both ommatidia. (**f3,4**) Ambiguities (?) suggest possible 5 in panel f3 or at least six profiles in panel f4. (**g1-4**) Interpretive diagrams of putative cone cells based on the brightest profiles in each ommatidium extending outwards to the level of the assumed crystalline cone (light gray) overlain by its lens (darker gray). Numbers indicate putative cone cells. (**column 5**) Intensity values at control area 5 in panel b. Scale bars: 50 µm in panel a; 20µm in panel c; 20µm in panel d for rows d-f.

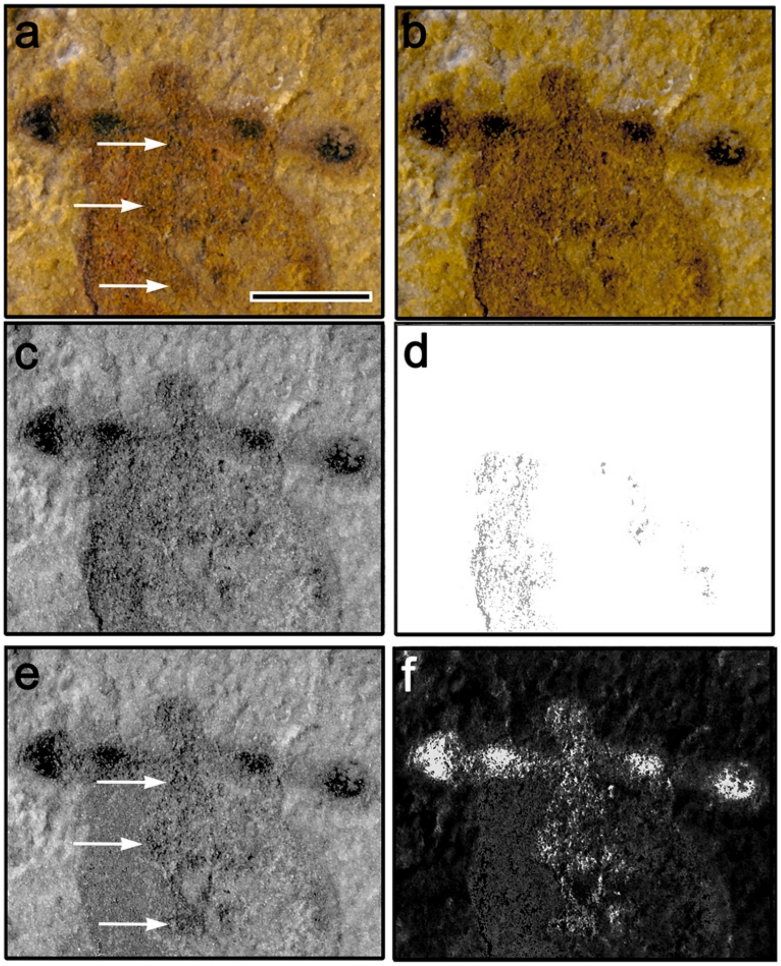

Supplementary Figure 3. Exposure of trace neuropils and tracts in specimen YKLP11367. (**a**) Photographic raw image reveals optic centers in the eyestalks and three approximately symmetric arrangements in the cephalon (arrowed in a) discernable despite color and density varying across the specimen. (**b, c**) Selective removal of the dominant orange tint further indicates fossilized soft tissue but accentuates imbalance. This is corrected by converting to grey-scale mode **(c)** and using the Adobe threshold functions to identify areas of the matrix above threshold levels **(d)** and subtract them from (c). **(e)** Gray levels are consequently balanced on the left and to the extreme right. (**f**) Black-white reversal provides the final profiles for tracing and reconstruction. Scale bar in panel a = 0.5mm

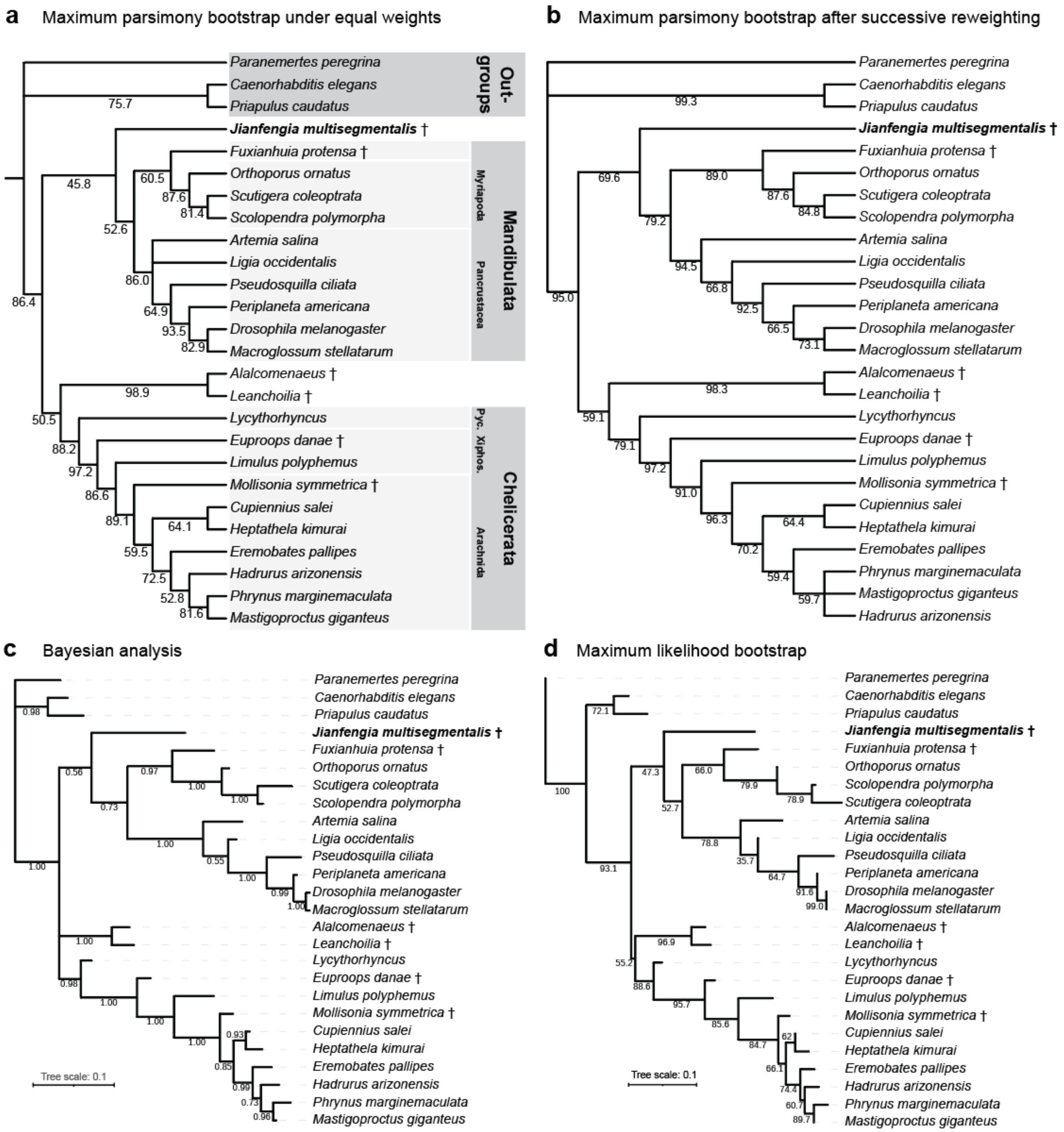

Supplementary Figure 4. Inferred evolutionary relationships support *Jianfengia* as sister to all Mandibulata. Summary of phylogenetic analyses of 120 characters applied to 17 living arthropod species, the Cambrian Burgess-Shale-Type fossils *Mollisonia symmetrica*, *Alalcomenaeus,* and *Leanchoilia,* the Chengjiang fossil *Fuxianhuia protensa,* the fossil limuliid *Euproops danae*, and out-groups: cycloneuralians *Caenorhabditis elegans* and *Priapulus caudatus*, and nemertean *Paranemertes peregrina*. Maximum parsimony bootstrap analyses were performed with 1,000 replicates on the data matrix in Supplementary Data 1 with either all characters under equal weights **(a)** or after successive reweighting provided a stable character

weight matrix **(b).** The tree in **(a)** represents the consensus tree of the two most parsimonious trees from a heuristic search (see Fig. 5) with support values at the nodes from the bootstrap analysis. **(c)** Markov Chain Monte Carlo (MCMC) Bayesian analysis was also performed on the data for 100 million generations under the Markov *k* (Mk) model and the first 25% of trees were removed for as a burn-in fraction. The resulting tree represents a majority rule consensus tree of post-burn-in trees with posterior probabilities at nodes. Maximum likelihood analysis was also performed as described in the text. The tree with the highest likelihood from a thorough heuristic search is shown **(d)** with support values from 1,000 bootstrap replicates. In each analysis there is support for *Jianfengia multisegmentalis* as the sister group to all Mandibulata. Pyc. = Pycnogonida; Xiphos. = Xiphosura; † = extinct taxon.

Supplementary Tables

Contents comprise:

Supplementary Table 1. Terminologies of euarthropod body partitioning and their underlying rationale.
Supplementary Table 2. Subtype-specific features classify Megacheira.

A-P axis partitioning	Segment term	Classic nomenclature	Genetic domain nomenclature	Associated appendage	Genetic code
1st segment/ neuromere		Protocerebrum	ce1 domain = prosocerebrum.<br>ce2 domain = protocerebrum	ce1 = labra<br>ce2 = eyestalks	• ce1=*FoxQ2-Six3-hbn*<br>• ce2=*Six3-Otx-Pax6*<br>• ce3=*Emx-Nk2-exd*<br>• Non-Hox<br>• Non-mesoderm
2nd segment/ neuromere	A1	Deutocerebrum	ce3 domain = deutocerebrum	ce3 = chelicerae (*sensu.lato*), antennules	
3rd segment/ neuromere	A2	Tritocerebrum	Trunk segment T1	biramous limb/ appendage*	• Hox code<br>• mesoderm-related
Trunk segment 1	A3	Mandibular gnathal segment/ neuromere	Trunk segment T2	biramous limb/ appendage*	
Trunk segment 2	A4	1st Maxillary gnathal segment/ neuromere	Trunk segment T3	biramous limb/ appendage*	
Trunk segment 3	A4	2nd Maxillary gnathal segment/ neuromere	Trunk segment T4	biramous limb/ appendage*	
Trunk segment 4	T1	1st trunk segment/ganglion	Trunk segment T5	biramous limb/ appendage	
Trunk segment 5	T2	2nd trunk segment/ganglion	Trunk segment T6	biramous limb/ appendage	
Trunk segment 6	T3	3rd trunk segment/ganglion	Trunk segment T7	biramous limb/ appendage	
Trunk segment 7	T4	4th trunk segment/ganglion	Trunk segment T8	biramous limb/ appendage	

Supplementary Table 1. Terminologies of euarthropod body partitioning and their underlying rationale. Various nomenclatures are used in naming parts of the brain, the segmented trunk, and their associated appendages with (*) segments T1-T4 ancestrally undifferentiated but divergently specialized in many stem lineages, such as the acquisition of mandible morphology of the T2 basipod[39-41]. Historical nomenclatures refer to traditional views of a segmented brain. However, the brain's embryonic formation is asegmental and mediated by a developmental program that is genetically distinct from that specifying the reiterated segmentation of the trunk. The genetic code underlying nervous system partitioning[4], provides the most parsimonious and biologically relevant terminology. Abbreviation: *sl., sensu lato*.

Genera	4 eyes, sessile or on short stalks	Rostral ocelli	Single pair of stalked compound eyes	GA pectinate with flagella	GA short, stout	Telson splayed or elongate	Telson paddle-like	Post-deutocerebral segments beneath carapace	Total post-deutocerebral segments	Resulting Group
Yohoia		?	X		X		X	3	16	1
Haikoucaris		?	X		X		X	3	16	
Tanglangia		?	X		X	elongate spine		3	16	
Jianfengia		X	X		X	elongate blade		3	25	2
Pseudoiulia		?	X		X	short spine?		3	34	
Fortiforceps		?	X		X	splayed fan		3	24	
Alalcomenaeus	X	–		X			X	3	14	3
Leanchoilia	X	–		X			X	3	14	

Supplementary Table 2. **Subtype-specific features classify Megacheira.** The paraphyletic euarthropod clade known as Megacheira is defined by its deutocerebral appendages, also called "great appendages" (GAs) composed of two proximal articles with an elbow-like joint continuing as 3-4 distal articles. Genera listed here exclude novel specimens known from single examples. Cross-comparison of species-specific features identifies three natural groups based on combinations of three traits.

GROUP 1 (*Yohoia, Haikoucaris*[37]*, Tanglangia*): three post-deutocerebral trunk segments obscured by carapace, 16 trunk segments total; single pair of stalked compound eyes; robust GAs with stout spines.

GROUP 2 (*Jianfengia, Pseudoiulia*, *Fortiforceps*[50]): three post-deutocerebral trunk segments wholly obscured by carapace, 24-34 trunk segments total; single pair of stalked compound eyes; spike-like distal articles of the stout GAs.

GROUP 3 (*Alalacomeneaus, Leanchoilia*)[35,36]: three post-deutocerebral trunk segments obscured by carapace, 14 trunk segments total; quartet of sessile, or short-stalked eyes; delicate GA with three pectinate distal articles each providing a prolonged flagellum.
