# Supplementary material for: Brain anatomy of the Cambrian fossil Jianfengia multisegmentalis informs euarthropod phylogeny
Source: Nat Commun. 2025 Aug 28;16:7938. doi: 10.1038/s41467-025-62849-w (PMC12394709; doi:10.1038/s41467-025-62849-w)
Supplement: Supplementary file 2 — Description of Addtional Supplementary Files [file 41467_2025_62849_MOESM2_ESM.pdf]

Description of Additional Supplementary Files

This file includes:
Supplmental Data 1: The matrix showing occurrence of traits (green) or their absence (blank) shown against taxa.
Supplemental Data 2: The Nexus file of character matrix used in all phylogenetic analyses.
