# Supplementary material for: Brain anatomy of the Cambrian fossil Jianfengia multisegmentalis informs euarthropod phylogeny
Source: Nat Commun. 2025 Aug 28;16:7938. doi: 10.1038/s41467-025-62849-w (PMC12394709; doi:10.1038/s41467-025-62849-w)
Supplement: Supplementary file 4 — Supplmentary Data 2 [file 41467_2025_62849_MOESM4_ESM.zip › Nexus/NEXUS.docx]

*#NEXUS*

*BEGIN DATA.*

*DIMENSIONS NTAX=26 NCHAR=120;*

*FORMAT DATATYPE=STANDARD MISSING=? GAP=- SYMBOLS = " 0 1";*

*MATRIX*

*Paranemertes_peregrina*

*1000000000000000000000001110000000000000000000000000000000100000000000*

*00000000000000000000000000000010000000001111100000*

*Orthoporus_ornatus*

*0110000000000000001010010000100001001101000010000000000000110000100001*

*10000000000000000001010010100000010001000000000000*

*Scutigera_coleoptrata*

*0111000000000000011010010000100001001101000010000000000010110010101000*

*10000011100000000011010000010000010001000000000000*

*Scolopendra_polymorpha*

*0111000000000000001010010000100001001101000010000000000000110010101000*

*10000011100000000001000010100000010001000000000000*

*Lycythorhyncus*

*0111010001010011000110000000000000010000000000000000000000000000000000*

*00000000000000000000000000000000000001000000000000*

*Limulus_polyphemus*

*0111010001110111100111000001010100110010011100100000000010000000000000*

*00000000001100001100000000000000101001100000000000*

*Hadrurus_arizonensis*

*0110101110100111100111000001010011010010011100100000000000000000000000*

*00000000010100100000000000000000101011000000000000*

*Eremobates_pallipes*

*0110101010110111100111000001010011010010011100100000000000000000000000*

*00000000010110000000000000000000100101000000000000*

*Mastigoproctus_giganteus*

*0110101010010111100111000001010011010010011100100000000000000000000000*

*00000000010100000000000000000000001011000000000000*

*Phrynus_marginemaculata*

*0110101010010111100111000001010011010010011100100000000000000000000000*

*00000000010101011000000000000000001011000000000000*

*Heptathela_kimurai*

*0110101110000111100111000001000010010010111100100000000000000000000000*

*00000000010100000000000000000000001001100000000000*

*Cupiennius_salei*

*0110101010110111100111000001000010010010111100100000000000000000000000*

*00000000010100000000000000000000001001100000000000*

*Ligia_occidentalis*

*0001010000001001011010010000100001000011000011010000010010000000100000*

*00100100000000000010100000010000010001000000000000*

*Pseudosquilla_ciliata*

*0001010000001001011011010000100001000011000011010100011100000000111000*

*01101111000000000011100100001000010001000000000000*

*13*

*Artemia_salina*

*0001010000001001011010010000000001000011000001010100010100000000110000*

*01000100000000000011100000010000010001000000011100*

*Periplaneta_americana*

*0001010000001001010010010000100001000101000010010100010010000000110000*

*00101111000000000010100100001000010001000000000000*

*Macroglossum_stellatarum*

*0001010000001001010010010000100001000101000010010100010010000000110000*

*00101111000000000010000100001001010001000000000000*

*Drosophila_melanogaster*

*0001010000001001010010010000100001000101000010010100010010000000110000*

*00101111000000000010000100001001010001000000000000*

*Fuxianhuia_protensa*

*0000000000001000010010010000000001000001000010000000001000110010100001*

*10000000000000000000000000100000010000000000110100*

*Jianfengia_multisegmentalis*

*0100010000001101011111110000000000000000000000001100010100000000000000*

*01010000000000000000000000001000010000010000111100*

*Leanchoilia*

*0110010000000101100010010000001100110000000000001010000000000000000000*

*00010000000000000100000000000000000100010000000011*

*Alalcomenaeus*

*0110010000000101000010000001001001010000000000001010000000000000000000*

*00010000000000000100000000000000100100010000000011*

*Mollisonia_symmetrica*

*0110101110110111100111000001000010010010011100100000000000000000000000*

*00000000000000000000000000000000101000100000000000*

*Euproops_danae*

*0110010001000010000111000001000100010010001100100000000010000000000000*

*00000000000000000000000000000000100000100000000000*

*Priapulus_caudatus*

*0010000000000000000000000010000000000000000000000001100001001101000100*

*00000000000000000000001001000010000001000010000000*

*Caenorhabditis_elegans*

*0010000000000000000000001110000000000000000000000001100000000100000010*

*00000000000000000000001001000110000001000100000000*

*;*

*END:*
