# Supplementary material for: Brain anatomy of the Cambrian fossil Jianfengia multisegmentalis informs euarthropod phylogeny
Source: Nat Commun. 2025 Aug 28;16:7938. doi: 10.1038/s41467-025-62849-w (PMC12394709; doi:10.1038/s41467-025-62849-w)
Supplement: Supplementary file 5 — Reporting Summary [file 41467_2025_62849_MOESM5_ESM.pdf]

nature portfolio

Corresponding author(s): Nicholas Strausfeld

Last updated by author(s): 7/26/2025

Reporting Summary

Nature Portfolio wishes to improve the reproducibility of the work that we publish. This form provides structure for consistency and transparency in reporting. For further information on Nature Portfolio policies, see our Editorial Policies and the Editorial Policy Checklist.

Statistics

For all statistical analyses, confirm that the following items are present in the figure legend, table legend, main text, or Methods section.

n/a	Confirmed	
☒	☐	The exact sample size (*n*) for each experimental group/condition, given as a discrete number and unit of measurement
☒	☐	A statement on whether measurements were taken from distinct samples or whether the same sample was measured repeatedly
☒	☐	The statistical test(s) used AND whether they are one- or two-sided<br>*Only common tests should be described solely by name; describe more complex techniques in the Methods section.*
☒	☐	A description of all covariates tested
☒	☐	A description of any assumptions or corrections, such as tests of normality and adjustment for multiple comparisons
☒	☐	A full description of the statistical parameters including central tendency (e.g. means) or other basic estimates (e.g. regression coefficient) AND variation (e.g. standard deviation) or associated estimates of uncertainty (e.g. confidence intervals)
☒	☐	For null hypothesis testing, the test statistic (e.g. *F*, *t*, *r*) with confidence intervals, effect sizes, degrees of freedom and *P* value noted<br>*Give P values as exact values whenever suitable.*
☐	☒	For Bayesian analysis, information on the choice of priors and Markov chain Monte Carlo settings
☒	☐	For hierarchical and complex designs, identification of the appropriate level for tests and full reporting of outcomes
☒	☐	Estimates of effect sizes (e.g. Cohen's *d*, Pearson's *r*), indicating how they were calculated

Our web collection on statistics for biologists contains articles on many of the points above.

Software and code

Policy information about availability of computer code

Data collection	Photographic data stored as digital photoshop files. No codes deployed. Data stored on portable hard drives, then downloaded as high resolution digital images on authors work computers and personal lap tops..
Data analysis	Documentation at various magnifications and illumination: ditectional, color filtered, polarized light, ultra violet illumination. Analysis of traits: Maximum parsimony and likelihood performed using PAUP* (Phylogenetic Analysis Using Parsimony*, version 4.0a168).

For manuscripts utilizing custom algorithms or software that are central to the research but not yet described in published literature, software must be made available to editors and reviewers. We strongly encourage code deposition in a community repository (e.g. GitHub). See the Nature Portfolio guidelines for submitting code & software for further information.

Data

Policy information about availability of data

All manuscripts must include a data availability statement. This statement should provide the following information, where applicable:

- Accession codes, unique identifiers, or web links for publicly available datasets
- A description of any restrictions on data availability
- For clinical datasets or third party data, please ensure that the statement adheres to our policy

See also Data availability explained in section "Reporting for specific materials, systems and methods.'
Cambrian fossils as research material are curated at the Yunnan Key Laboratory for Paleobiology (YKLP). Availability is dependent on the staff member (in the

present instance, the YKLP Director, Professor Xianguang Hou) involved in the combinatorial research planned by the research team.
The following accession codes (unique identifiers) denote the fossil material of exclusively Jianfengia multisegmentalis used for this publication. These accession numbers are as follows: YKLP11117, YKLP11367, YKLP 11368, YKLP 11369, YKLP17299 and NIGPAS 100123b are curated at the Yunnan Key Laboratory for Palaeobiology (YKLP), Institute of Paleontology, Yunnan University, Yunnan, Kunming, China.
To the best of my (NJS) my knowledge once study material is selected for onsite observation there are no formal restrictions of data availabilty.
No clinical data was used for any of the research described in this account.

Research involving human participants, their data, or biological material

Policy information about studies with human participants or human data. See also policy information about sex, gender (identity/presentation), and sexual orientation and race, ethnicity and racism.

Reporting on sex and gender	none reported
Reporting on race, ethnicity, or other socially relevant groupings	Research collaborators are male Asian and male caucasian reseachers.
Population characteristics	Not assessed for this study
Recruitment	No personnel recruitment for this study
Ethics oversight	Ethics oversight was maintained by mutual interaction/oversight by members of the research team, namely the present co-authors.

Note that full information on the approval of the study protocol must also be provided in the manuscript.

Field-specific reporting

Please select the one below that is the best fit for your research. If you are not sure, read the appropriate sections before making your selection.

☐ Life sciences ☐ Behavioural & social sciences ☒ Ecological, evolutionary & environmental sciences

For a reference copy of the document with all sections, see nature.com/documents/nr-reporting-summary-flat.pdf

Ecological, evolutionary & environmental sciences study design

All studies must disclose on these points even when the disclosure is negative.

Study description	Analysis of fosilized nervous tissue in mid-Cambrian Euarthropods
Research sample	Six specimens of Jianfengia multisegmentalis (Hou) curated at the Yunnan Key Laboratory of Paleobiology.
Sampling strategy	Direct microscopical observations.
Data collection	Data (fossils) collect by locally recruited helpers at the quarry site under the supervision of Prof. Hou. Material cleaned and trimmed in the YKLP research lab. Data (candidate fossils) selected fo further study under the microscope. Digital photographs then accrued for on-site analysis and discussion.
Timing and spatial scale	Photographs of specimens obtained at the YKLP during reoccurring visits by NJS during 2014-2019. Total of 4 research visits consisted of 7-14 working days
Data exclusions	No data excluded
Reproducibility	The data, here meaning documentation, photogrphy, was on unique fossil specimens.
Randomization	No radomizing applicable. to studying fossil material
Blinding	Blinding inapplicable in studying fossil material/morphologies

Did the study involve field work? ☐ Yes ☒ No

Reporting for specific materials, systems and methods

We require information from authors about some types of materials, experimental systems and methods used in many studies. Here, indicate whether each material, system or method listed is relevant to your study. If you are not sure if a list item applies to your research, read the appropriate section before selecting a response.

Materials & experimental systems

n/a	Involved in the study
☒	☐ Antibodies
☒	☐ Eukaryotic cell lines
☐	☒ Palaeontology and archaeology
☒	☐ Animals and other organisms
☒	☐ Clinical data
☒	☐ Dual use research of concern
☒	☐ Plants

Methods

n/a	Involved in the study
☒	☐ ChIP-seq
☒	☐ Flow cytometry
☒	☐ MRI-based neuroimaging

Palaeontology and Archaeology

Specimen provenance

Specimens collected at officially approve quarries in the vicinity of the city of Kunming. All material was curated and stored at the YKLP, Kunming, China where they recieved accession numbers an placed in designated folders

Specimen deposition

Specimens reside at the Yunnan Key laboratory for Paleobiology, (YKLP), Kunming, China.

Dating methods

Specimens collected from from the Cambrian (Series 2, Stage 3) Eoredlichia–Wutingaspis trilobite biozone,
39 Yu'anshan Member, Chiungchussu Formation. These dating have been obtained by local expertise of trained geologists with expertise of local sections.

☒ Tick this box to confirm that the raw and calibrated dates are available in the paper or in Supplementary Information.

Ethics oversight

Approved by the then director (through 2019) of the YKLP who is also a co-author on the present paper.

Note that full information on the approval of the study protocol must also be provided in the manuscript.

Plants

Seed stocks

No plants used in this research

Novel plant genotypes

See above

Authentication

See above
